# Existential and Mindfulness–Based Intervention to Increase Self-Compassion in Apparently Healthy Subjects (the EXMIND Study): A Randomized Controlled Trial

**DOI:** 10.3389/fpsyt.2019.00538

**Published:** 2019-08-02

**Authors:** Akari Sakai, Takeshi Terao, Nobuko Kawano, Mari Akase, Koji Hatano, Masanao Shirahama, Hirofumi Hirakawa, Kentaro Kohno, Ayako Inoue, Nobuyoshi Ishii

**Affiliations:** Department of Neuropsychiatry, Faculty of Medicine, Oita University, Yufu, Japan

**Keywords:** psychotherapy, mindfulness, existential approach, self-compassion, randomized controlled trial

## Abstract

**Objectives:** Mindfulness is a method of training the regulation of attention with non-judgmental acceptance that is linked to beneficial effects on health. The existential approach supports the uniqueness of each individual and helps to provide meaning to their lives. In this randomized controlled trial, we examined whether mindfulness-based intervention (MBI) and the existential approach could be combined sequentially and whether they operated antagonistically or cooperatively.

**Methods:** One hundred thirty-seven participants aged 20 years or older without any severe mental disorders were randomly assigned (1:1), *via* an envelope method, to receive either 8-week MBI (N = 68) or 4-week MBI followed by 4-week existential approach (EXMIND) (N = 69). Participants were first allocated to a waiting-list group and subsequently randomized to the MBI group or EXMIND group. The primary outcome was self-compassion scale (SCS) total scores at 0, 4, and 8 weeks during intervention or waiting. The analyses were performed by linear mixed models for both primary and secondary outcomes following the intention-to-treat principle.

**Results:** Both MBI and EXMIND groups had significantly increased SCS total scores compared to those of the waiting group, with mean SCS total scores of 2.3 (SD 3.0) in the MBI group and 2.1 (2.9) in the EXMIND group *versus* 0.3 (2.2) in the waiting group.

**Conclusions:** Our findings suggest that MBI followed by existential approach are not antagonistic and may have cooperative effects, suggesting that EXMIND may be a useful treatment.

## Introduction

Mindfulness is the process of acknowledging subjective experience, and it has emotional regulation process, which is broader than attentional control (1). Although attention is a key component of mindfulness practice, mindfulness also incorporates an openness to experience, which reflects a non-judgmental acceptance strongly linked to improved health (2). Mindfulness-based interventions (MBIs) can be traced back to the late 1970s. A mindfulness-based stress reduction (MBSR) program was begun in 1979 in the basement of the University of Massachusetts Medical Center (3), where Kabat-Zinn (4) initially reported that mindfulness meditation produced significant pain reduction in chronic pain patients. Since then, numerous treatment protocols based on MBSR, such as mindfulness-based cognitive therapy (MBCT), have been developed. MBSR and MBCT are two of the most widely used MBIs (5). Their positive effects on mental health and quality of life have been reported in diverse clinical and non-clinical populations (6–8).

In contrast, existential therapies can be defined as psychological interventions that are informed, to a significant extent, by the teaching of existential philosophers, most notably Heidegger, Sartre, Buber, Tillich, Kierkegaard, and Nietzsche (9). Vos et al. (10) reviewed “four main schools have been identified in the existential therapies field: 1) Daseinsanalysis where patients are provided with a permissive therapeutic relationship in which they can express themselves freely and develop greater openness toward their world; 2) meaning or logo-therapies which aim to help clients establish meaning and purpose in their lives; 3) a British school of existential therapy which adopts a primarily descriptive, phenomenological stance, with clients encouraged to explore their lived experiences; and 4) the existential–humanistic approach to help clients face the ultimate givens of life, in particular, mortality, freedom, isolation, and meaninglessness.” Heidegger, in his book entitled “Sein und Zeit” (11) which was translated to English version “Being and Time” (12), stated “When resoluteness, anticipating, has caught up with the possibility of death in its potentiality-of-being, the authentic existence of Dasein can no longer be overtaken by anything” (12). That is, “death awareness and contemplating it allows one to prioritize and decide what is important to a meaningful and creative experience for each individual life” (13). Although Heidegger did not write directly about the work of therapists, Orbanic (14) described how therapists have used Heidegger’s philosophy and summarized as follows: “The therapist assists distraught individuals in discovering meaningfulness, finding purpose, and actualizing self-love by facilitating deeper self-awareness, appreciation, and understanding of who they are, have been and their unique process of becoming. The therapist, guided by this view, helps a hopeless individual realize that as a ‘person being-in-time,’ he or she is always living in limitless temporality where change is inevitable, and the future bestows hope.” Similarly, Moore and Goldner-Vukov (15) suggest “an existential approach is needed to comprehend the wholeness of patients. Existential phenomenology supports the uniqueness of each individual and helps guide clinicians in establishing a respectful therapeutic relationship that is supportive of the recovery approach and the self-determination of patients.”

We thought that MBI has static or passive components such as attentional control and non-judgmental acceptance, whereas the existential approach has dynamic or active components such as discovering meaningfulness, finding purpose, and actualizing self-esteem. In other words, it seems likely that MBI tries to accept the world as it is whereas existential approach attempts changing the world within each individuals. Therefore, in our opinion, these two therapies may have opposite components and it is unclear whether combining MBI and existential approach sequentially would cause antagonistic effects or whether they operate in a cooperative manner. We investigated self-compassion and depression because self-compassion seems to be one of common targets for both mindfulness and existential approach, which was measured with the Self-Compassion Scale-Japanese version (SCS-J) (16) consisting of six subscales (self-kindness, common humanity, mindfulness, self-judgment, isolation, and over-identification), which were considered to be factors associated with MBI and existential approach. In addition, we measured depression to clarify whether MBI and/or existential approach directly improve self-compassion or indirectly do so *via* improving depression. In the present study, we examined these issues in a randomized controlled trial.

## Materials and Methods

### Study Population

We performed this randomized controlled trial at the city hall of Oita city (Horuto-Hall Oita), Japan. Eligibility criteria were 20 years or older and provision of written informed consent. We recruited participants *via* an electronic bulletin board, bulletin board, and fly sheet. We excluded participants if they suffered from serious psychiatric disorders. We performed the Mini-International Neuropsychiatric Interview (M.I.N.I.) for all participants to examine recent or past psychiatric history. Participants were recruited from Oct 1, 2016, and June 30, 2018. The Institutional Review Board of Oita University Faculty of Medicine approved the trial on Sep 14, 2016 (number B16-023). All participants provided written informed consent.

### Randomization and Masking

**Figure 1:**
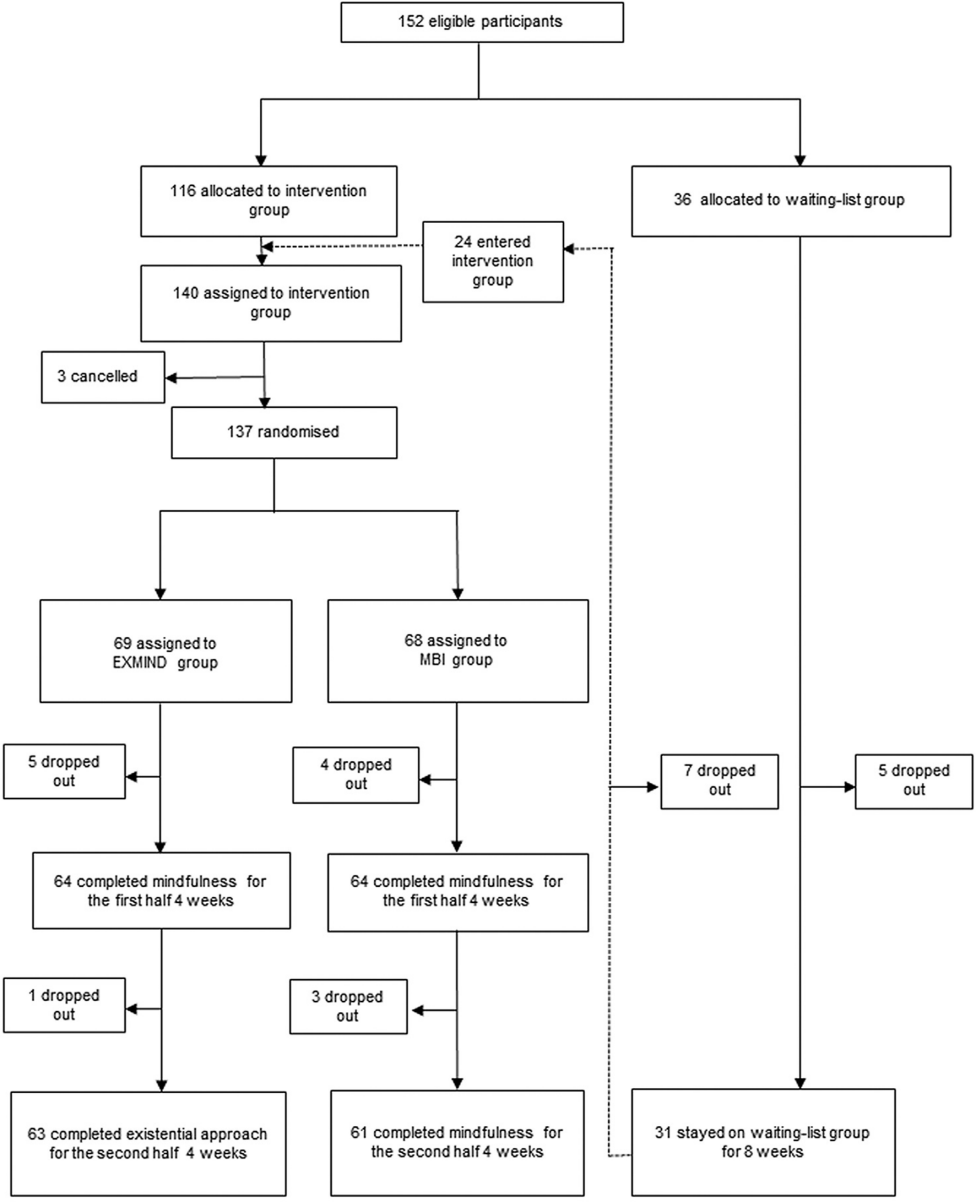
CONSORT flow chart. Participants were randomly assigned (1:1) to receive either 8-week mindfulness-based intervention (MBI) or sequential 4-week mindfulness-based intervention plus 4-week existential approach (EXMIND).

Every few months, participants were assigned to the intervention group or waiting-list group (4:1). The new intervention group and participants from the waiting-list group who had been waiting for more than 8 weeks were combined as the intervention group. As shown in **Figure 1** (CONSORT flow chart), they were randomly assigned to either the 8-week MBI group or 4-week MBI plus 4-week existential approach (EXMIND) group (1:1) *via* an envelope method which was performed by a third party external to this study. Participants received the explanation that the MBI group would further master the skill of mindfulness while the EXMIND group would combine an existential approach with mindfulness.

Each participant was automatically informed of their allocation after completion of the baseline questionnaire. Concurrently, actual working members of the study were automatically informed of participants’ allocation. The allocation process was concealed from the researchers involved in statistical analysis. The researchers performing analyses had never visited the Horuto-Hall Oita to see the participants, keeping blind to the situation. The data of all participants were anonymized.

### Study Design

Our intervention consisted of lectures and the practice of mindfulness and existential approach. Actual working members of the study consisted of a psychiatrist and two psychologists. All of them had much meditation experience for several years, and two of them had undertaken a mindfulness course at the Japanese Association of Mindfulness. Also, all had much experience of existential approach in routine clinical settings.

MBI and EXMIND consisted of weekly MBI for 8 weeks or weekly MBI for 4 weeks plus subsequent weekly existential approach for 4 weeks, respectively. Both interventions comprised eight sessions for 8 weeks. The maximum number of participants per session was up to 23. It took 90 to 120 min for participants to complete each session. Just before each session, 30 min was allocated for them to fill out the questionnaires. No payment was required from participants.

**Figure 2:**
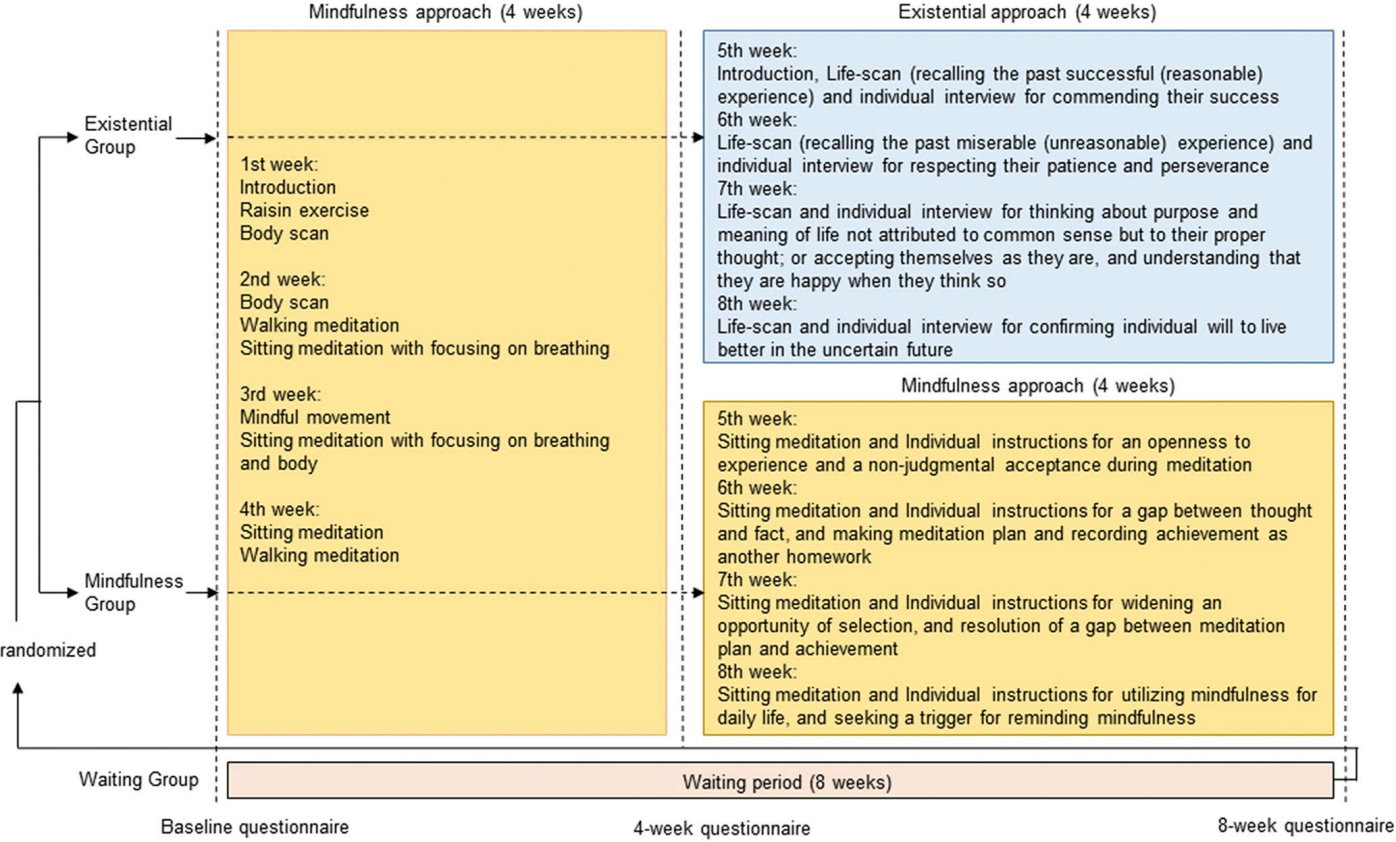
Content of intervention. MBI and EXMIND consisted of weekly mindfulness-based intervention for 8 weeks or weekly mindfulness-based intervention for 4 weeks followed by weekly existential approach for 4 weeks, respectively. Both interventions comprised eight sessions for 8 weeks.

As shown in **Figure 2**, during the first half (4 weeks), both the MBI and EXMIND groups attended the same MBI sessions which included raisin exercise, mindful breathing, body scan, walking meditation, and sitting meditation. Participants received explanatory notes at every session. Homework including both formal and informal trainings was encouraged for participants to train themselves for mindfulness reskilling.

During the second half (4 weeks), the MBI group continued to perform meditation and homework, and thereafter discussed their experiences during individual instruction; at session 5, sitting meditation and individual instructions for an openness to experience and non-judgmental acceptance during meditation; at session 6, sitting meditation and individual instructions for a gap between thought and fact, making a meditation plan, and recording achievement as homework; at session 7, sitting meditation and individual instructions for widening opportunities for selection, and resolution of the gap between meditation plan and achievement; and at session 8, sitting meditation, individual instructions for utilizing mindfulness for daily life, and seeking triggers to remind of mindfulness.

During the second half (4 weeks), the EXMIND group started life-scan where participants observed their life from the past, present, and future. This method was based on aforementioned description of “The therapist assists distraught individuals in discovering meaningfulness, finding purpose, and actualizing self-love by facilitating deeper self-awareness, appreciation, and understanding of who they are, have been and their unique process of becoming. The therapist, guided by this view, helps a hopeless individual realize that as a ‘person being-in-time,’ he or she is always living in limitless temporality where change is inevitable, and the future bestows hope” (14), which was based on Heidegger’s philosophy. In order to do so, we performed a life-scan where individuals were instructed to recall past successful (reasonable) experiences and their success was commended by actual working members, which probably brought about meaningfulness and pleasure of achieving purpose. Also, individuals were asked to recall past miserable (unreasonable) experiences, and their patience and perseverance were respected by the actual working members, which probably actualized self-love (or self-esteem). Moreover, thinking about the purpose and meaning of life not attributed to common sense but to their individual proper thought during individual interviews was considered to be the very existential, and considering that death awareness and contemplating it allow one to prioritize and decide what is important to a meaningful and creative experience for each individual life (13); confirming individual will to live better in the uncertain future during individual interviews was likely to be the very existential.

Following the above notions, in brief, they continued to perform mindfulness as homework where they could include life-scan; at session 5, recalling past successful (reasonable) experiences and their success was commended during individual interviews; at session 6, recalling past miserable (unreasonable) experiences, and their patience and perseverance were respected during individual interviews; at session 7, thinking about the purpose and meaning of life not attributed to common sense but to their proper thought, accepting themselves as they are, and understanding that they are happy when they think so during individual interviews; and at session 8, confirming individual will to live better in the uncertain future during individual interviews.

We call this study and ongoing studies “The EXMIND studies” in order to emphasize the importance of the combination of existential approach and mindfulness, one of which initially investigated the effects of MBI and EXMIND (this study). Ongoing studies are investigating the factors predicting response to MBI or EXMIND, and a follow-up study.

### Outcomes

The primary outcome was self-compassion total scores because self-compassion seems to be one of common targets for both mindfulness and existential approach, which was measured with the Self-Compassion Scale-Japanese version (SCS-J) (16) at baseline, 4 weeks, and 8 weeks. Although this scale has six subscales (self-kindness, common humanity, mindfulness, self-judgment, isolation, and over-identification), a total SCS score can be used as an overall measure of self-compassion (17) but six subscale scores (representing the constituent components of self-compassion) have been reported not to represent compassionate and uncompassionate self-responding (18). Despite this limitation, the subscale scores were used as a secondary outcome. Also, depressive state, measured with the Beck Depression Inventory (BDI) at baseline, 4 weeks, and 8 weeks, was used as a secondary outcome.

### Statistical Analysis

To detect changes in SCS-J score by MBI or EXMIND, considering the effect size of MBI may be 0.5 at a P-value of less than 0.05 with 80% power; 60 participants per group were estimated to be needed, allowing for 20% loss to follow-up.

To investigate significant changes over time, linear mixed models (LMM) were conducted for both primary and secondary outcomes following the intention-to-treat principle. A two-sided P-value of less than 0.05 was considered to be statistically significant. The use of LMM provided the means to include participants with incomplete data to assess the treatment effect over time (i.e., group × time interaction). In our models, intervention group, time, and the interactions between the intervention groups and time were treated as fixed factors. The association between changes in primary and secondary outcomes, which were calculated by subtracting SCS scores at baseline from those at 8 weeks and by subtracting BDI scores at 8 weeks from those at baseline, was examined by Pearson’s coefficient. Statistical analyses of the primary and secondary outcome measures were conducted in SPSS version 25.

The study protocol was submitted to UMIN on May 5, 2017, and accepted on May 29, 2017. This trial was registered as “A comparison of mindfulness therapy with existential approach and without: randomized controlled study in healthy volunteers” in University hospital Medical Information Network (UMIN), number UMIN000027537. This procedure was assessed by Supporting Doc 1.

## Results

Between Oct 1, 2016, and June 30, 2018, we assigned 152 participants to the intervention group (n = 116) and the waiting-list group (n = 36). 12 of the waiting-list group dropped out. Of the waiting-list group, 24 waiting completers entered the intervention group (n = 140). Excluding three cancellers, 137 participants were randomized to the EXMIND group (n = 69) or the MBI group (n = 68). Six of the EXMIND group and seven of the MBI group dropped out. 63 (91.3%) participants completed the EXMIND whereas 61 (89.7%) participants completed the MBI. The demographic characteristics of participants are presented in **Table 1**. The mean age of participants was 49.4 (SD 12.5). The majority of participants were women (82.6%). Although the participants were apparently healthy, M.I.N.I. revealed that one third of participants had recent or past psychiatric history. Also, the mental state of the participants was assessed using Hamilton Rating Scale for Depression (HRSD) and Young Mania Rating Scale (YMRS) as well as BDI. There were no significant differences in these factors between groups.

**Table 1:** Participant demographics.^†^

	EXMIND group (*n* = 63)	MBI group (*n* = 61)	Waiting group (*n* = 31)	*P*
Age	48.4 (12.6)	49.6 (11.6)	51.1 (14.1)	0.61
Sex				0.97
Male	11 (17%)	11 (18%)	5 (16%)	
Female	52 (83%)	50 (82%)	26 (84%)	
Employment				0.98
Full-time employed	36 (57%)	34 (56%)	15 (48%)	
Part-time employed	8 (13%)	7 (11%)	6 (19%)	
Self employed	4 (6%)	3 (5%)	1 (3%)	
Unemployed	13 (21%)	15 (25%)	8 (25%)	
Student	2 (3%)	2 (3%)	1 (3%)	
Education				0.53
High school	6 (10%)	11 (18%)	3 (10%)	
Junior/vocational college	28 (44%)	24 (39%)	11 (35%)	
Bachelor/Master/doctorate	29 (46%)	26 (43%)	17 (55%)	
Marital status				0.72
Unmarried	18 (29%)	17 (28%)	10 (32%)	
Married	35 (55%)	35 (57%)	16 (52%)	
Divorce	7 (11%)	7 (11%)	3 (10%)	
Bereavement	1 (2%)	1 (2%)	2 (6%)	
Others	2 (3%)	0 (0%)	0 (0%)	
No response	0 (0%)	1 (2%)	0 (0%)	
M.I.N.I.				0.17
(-)^‡^	44 (70%)	36 (59%)	24 (77%)	
(+)^§^	19 (30%)	25 (41%)	7 (23%)	
HRSD	2.2 (2.5)	2.6 (3.2)	2.0 (2.1)	0.54
YMRS	0.3 (0.8)	0.4 (0.9)	0.1 (0.3)	0.14
BDI	6.9 (6.4)	8.5 (8.1)	8.5 (5.1)	0.39
SCS				
Total	18.6 (4.4)	18.2 (4.0)	17.7 (3.6)	0.63
Self-kindness	3.2 (0.9)	3.1 (0.9)	3.0 (0.7)	0.65
Self-judgment	2.9 (0.9)	2.9 (0.9)	3.2 (0.8)	0.32
Common humanity	3.0 (0.9)	2.7 (0.8)	3.0 (0.8)	0.32
Isolation	2.6 (0.9)	2.6 (1.0)	2.8 (0.9)	0.84
Mindfulness	3.3 (0.8)	3.3 (0.8)	3.2 (0.8)	0.74
Over-identification	3.4 (1.0)	3.4 (1.0)	3.6 (0.8)	0.53

^†^Data are mean (SD) or number (%) unless otherwise indicated.

^‡^(-) indicates no recent or past psychiatric history.

^§^(+) indicates recent or past psychiatric history.

### Primary Outcome Measures

**Figure 3:**
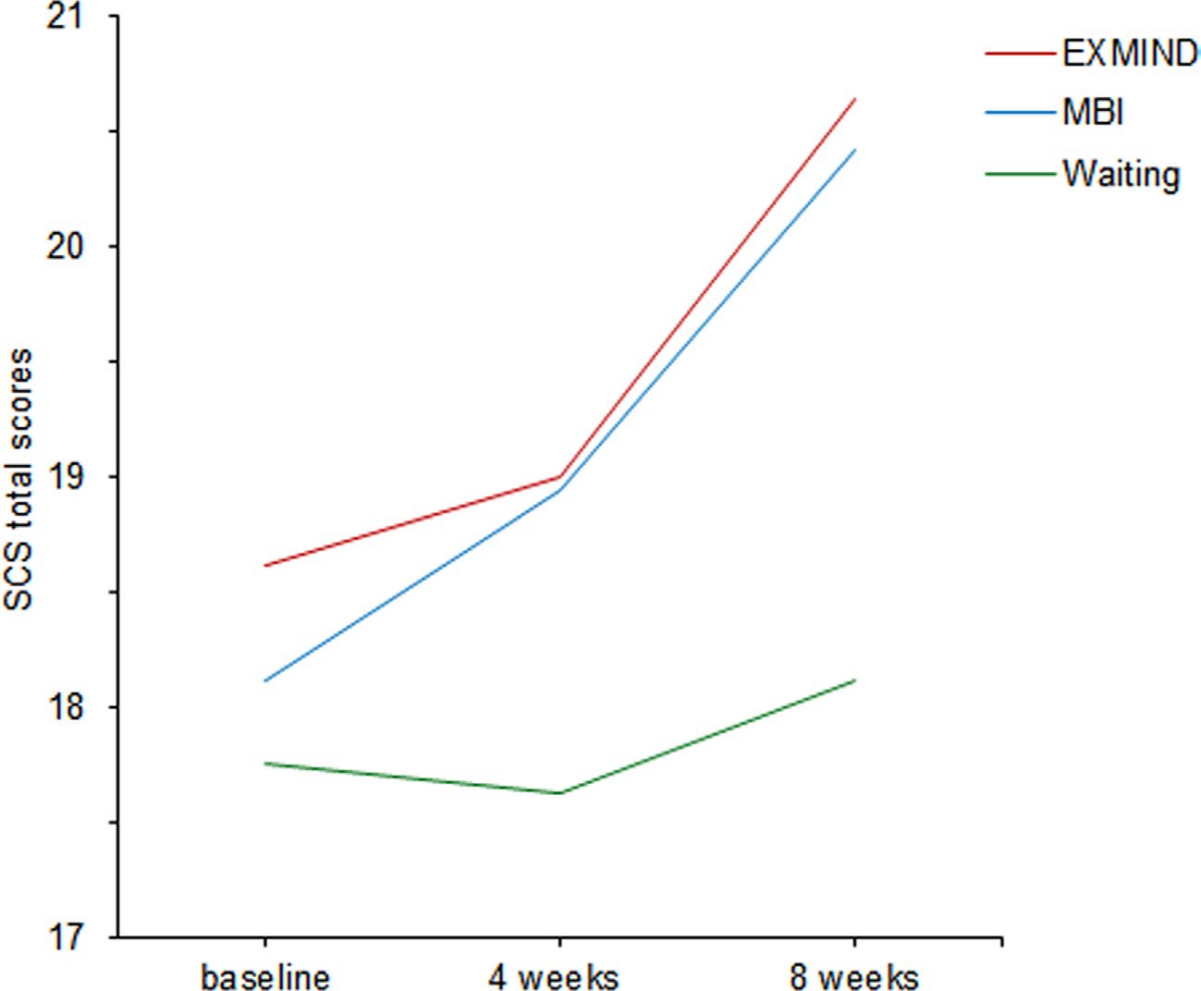
Changes from baseline to 8 weeks in SCS total scores of EXMIND, MBI, and waiting groups. SCS, Self-Compassion Scale.

The SCS total scores in both EXMIND and MBI groups were significantly increased at 8 weeks after baseline assessment compared to those of the waiting group. The estimated means as generated by the LMM procedure were used to produce the trajectories in **Figure 3**. Overall, LMM demonstrated a significant group × time interaction [F(4,291) = 2.75, P = 0.028]. At 8 weeks after baseline assessment, a significant relative change in score was revealed between EXMIND and waiting groups, and between MBI and waiting groups.

### Secondary Outcome Measures

**Figure 4:**
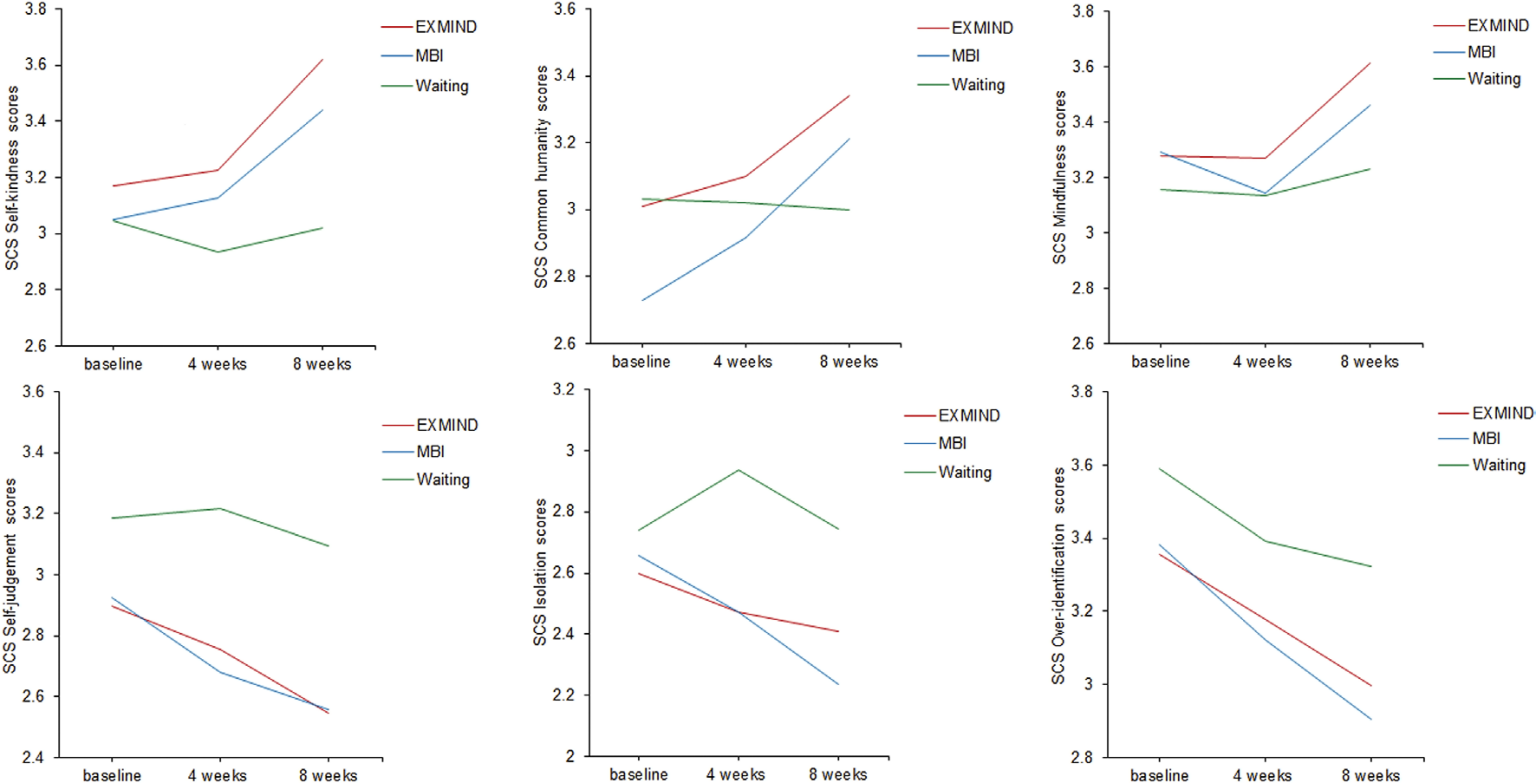
Changes from baseline to 8 weeks in SCS subscale scores of EXMIND, MBI, and waiting groups.

The SCS self-kindness subscale scores in both EXMIND and MBI groups were significantly increased at 8 weeks after baseline assessment compared to those of the waiting group. The estimated means as generated by LMM procedure were used to produce the trajectories in **Figure 4**. Overall, LMM demonstrated a significant group × time interaction [F(4,294) = 2.84, P = 0.024]. At 8 weeks after baseline assessment, a significant relative change of score was revealed between the EXMIND and waiting groups but not between the MBI and waiting groups. There were no significant differences between intervention groups and waiting groups in the other five subscales.

**Figure 5:**
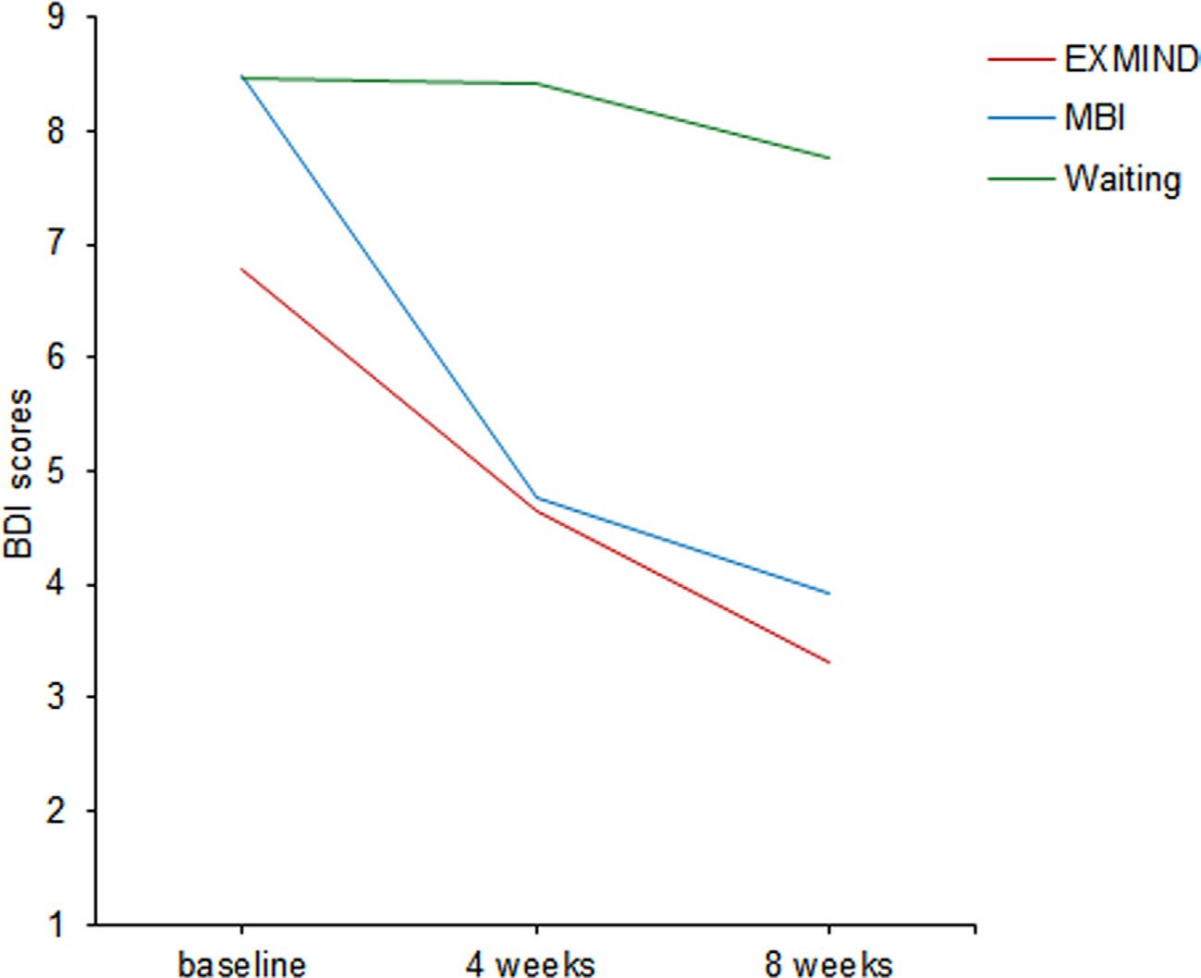
Changes from baseline to 8 weeks in BDI scores of EXMIND, MBI, and waiting groups. BDI, Beck Depression Inventory.

The BDI scores in both EXMIND and MBI groups were significantly decreased at 4 and 8 weeks after baseline assessment compared to those of the waiting group. The estimated means as generated by the LMM procedure were used to produce the trajectories in **Figure 5**. Overall, LMM demonstrated a significant group × time interaction [F(4,262) = 3.72, P = 0.006]. At 4 and 8 weeks after baseline assessment, significant relative changes in scores were revealed between EXMIND and waiting groups and between the MBI and waiting groups.

### Associations Between Changes in Primary and Secondary Outcomes

Changes in SCS total scores were significantly and positively associated with changes in SCS self-kindness scores (r = 0.67, p < 0.0001), but not with changes in BDI scores (r = 0.069, p = 0.45).

### Additional Analysis

Because a third of apparently healthy participants had recent or past psychiatric history, we analyzed the SCS total scores of the participants with and without a psychiatric history, separately. As a result, LMM demonstrated a significant group × time interaction [F(4,190) = 2.65, P = 0.035] where both EXMIND and MBI groups were significantly better than waiting group in the participants without a psychiatric history, but there was no significant group × time interaction in the participants with a psychiatric history [F(4,95) = 2.05, P = 0.093].

## Discussion

Our findings showed that EXMIND and MBI groups achieved comparable effects on SCS total scores, SCS self-kindness scores, and BDI scores compared to those of the waiting group, suggesting that combined 4-week MBI followed by 4-week existential approach work cooperatively. The EXMIND and MBI groups continued to perform mindfulness homework during the second half. Therefore, the EXMIND group performed mindfulness at four sessions and at home during the first half, and existential approach at four sessions and mindfulness at home during the second half. On the other hand, the MBI group performed mindfulness at eight sessions and at home throughout the 8 weeks. As such, the existential approach was performed in the setting of mindfulness. If there was no effect of existential approach on SCS total scores, the MBI group may have improved more than the EXMIND group did, because the EXMIND group only performed mindfulness at home whereas the MBI group performed both at four sessions and at home. Therefore, comparison of the changes in SCS total scores suggests that the existential approach may be effective and additive to the effects of continuing mindfulness as homework.

The drop-out rate was 12 of 36 (33.3%) at waiting, 6 of 69 (8.6%) during EXMIND, and 7 of 68 (10.3%) during MBI, suggesting that one of three participants could not wait until intervention and one of 10 participants dropped out during intervention. This means that both therapies may be well-tolerated psychotherapy.

Although MBI and EXMIND may have opposite components [i.e., MBI tries to accept the world as it is (i.e., passive) whereas existential approach attempts changing the world (i.e., active) within each individuals] and our concern was whether combining MBI and existential approach sequentially would cause antagonistic effects, but, honestly speaking, we initially conjectured that the existential approach could induce more prominent synergistic effects in the setting of mindfulness in contrast to mindfulness alone because we expected that achieving attentional control and non-judgmental acceptance during mindfulness and subsequently discovering meaningfulness, finding purpose, and actualizing self-esteem may have improved mental health. Nevertheless, the present findings did not support this proposition but provided support that they do work cooperatively.

Changes in SCS total scores were positively associated with changes in SCS self-kindness scores but not with changes in BDI scores, suggesting that changes in self-compassion total scores may be due to changes in self-kindness scores, but not with changes in depressive state. There is, however, a report showing that improvements in mindfulness and self-compassion were significantly correlated with decreases in depression and anxiety (19). The discrepancy may be due to the fact that their subjects were depressive patients. Self-kindness of SCS is particularly related to self-kindness in unhappy situations; this was expressed in the sentences of questionnaires of SCS self-kindness. This is probably at least partially attributable to non-judgmental acceptance in the MBI group, and to recalling the past miserable (unreasonable) experience where participants were encouraged to commend their efforts and bearing of hardship in the EXMIND group. As shown in **Figure 4**, the time courses of SCS self-judgment, isolation, and over-identification scores suggested superiority of EXMIND and MBI groups to the waiting group, although the differences were not statistically significant.

As an additional analysis, there was a significant improvement of the SCS total scores in the participants without a psychiatric history (N = 104) where both EXMIND and MBI groups were significantly better than waiting group, but not in the participants with a psychiatric history (N = 51). This non-significance (P = 0.093) was probably due to the small number of the participants and not due to the ineffectiveness of EXMIND or MBI in the participants with a psychiatric history.

A limitation of this study is the lack of a 4-week existential approach plus MBI group and an 8-week existential approach group. Without such intervention, we do not know if the positive outcome of the combined group depends on the existential approach *per se*. Moreover, the allocation to waiting and intervention groups was not randomized. Nonetheless, the allocation to EXMIND and MBI groups was randomized, and both groups were adequately compared with ITT analyses. Another limitation is that M.I.N.I. revealed one third of apparently healthy participants had recent or past psychiatric history. As such, the present findings should be restricted to apparently healthy individuals with or without recent or past psychiatric history.

In conclusion, the present findings suggest that sequential MBI and existential approach may be cooperative, pointing to the therapeutic potential of EXMIND.

## Data Availability

The datasets generated for this study are available on request to the corresponding author.

## Ethics Statement

The Institutional Review Board of Oita University Faculty of Medicine approved the trial on Sep 14, 2016 (number B16-023). All participants provided written informed consent.

## Author Contributions

AS, TT, NK, and MA planned this study. AS, NK, and MA were actual working members. KH, MS, HH, KK, AI, and NI discussed the results with the actual working members. AS and TT mainly wrote the manuscript; the other authors supported the study and checked the manuscript. All authors read and approved the final manuscript.

## Conflict of Interest Statement

The authors declare that the research was conducted in the absence of any commercial or financial relationships that could be construed as a potential conflict of interest.

## Abbreviations Lists

BDI, Beck Depression Inventory; HRSD, Hamilton Rating Scale for Depression; M.I.N.I., Mini-International Neuropsychiatric Interview; SCS, Self-Compassion Scale; YMRS, Young Mania Rating Scale.

## References

[1] Cooper D Yap K Batalha L Mindfulness-based interventions and their effects on emotional clarity: a systematic review and meta-analysis. J Affect Disord. (2018) 235:265–76. 10.1016/j.jad.2018.04.018 29660642

[2] Keng SL Smoski M Robins C Effects of mindfulness on psychological health: a review of empirical studies. Clin Psychol Rev (2011) 31:1041–56. 10.1016/j.cpr.2011.04.006 PMC3679190 21802619

[3] Cullen M Mindfulness-based interventions: an emerging phenomenon. Mindfulness (2011) 2:186–93. 10.1007/s12671-011-0058-1

[4] Kabat-Zinn J An outpatient program in behavioral medicine for chronic pain patients based on the practice of mindfulness meditation: theoretical considerations and preliminary results. Gen Hosp Psychiatry (1982) 4:33–47. 10.1016/0163-8343(82)90026-3 7042457

[5] O’Leary K O’Neill S Dockray S A systematic review of the effects of mindfulness interventions on cortisol. J. Health Psychol. (2016) 21:2108–21. 10.1177/1359105315569095 25673371

[6] Khoury B Lecomte T Fortin G Masse M Therien P Bouchard V Mindfulness-based therapy: a comprehensive meta-analysis. Clin Psychol Rev (2013) 33:763–71. 10.1016/j.cpr.2013.05.005 23796855

[7] Goyal M Singh S Sibinga EM Gould NF Rowland-Seymour A Sharma R Meditation programs for psychological stress and well- being: a systematic review and meta-analysis. JAMA Intern Med (2014) 174:357–68. 10.1001/jamainternmed.2013.13018 PMC4142584 24395196

[8] Demarzo MMP Montero-Marin J Cuijpers P Zabaleta-del-Olmo E Mahtani KR Vellinga A The efficacy of mindfulness-based interventions in primary care: a meta-analytic review. Ann Fam Med (2015) 13:573–82. 10.1370/afm.1863 PMC4639383 26553897

[9] Cooper M The existential counseling primer: a concise, accessible and comprehensive introduction. London, England: PCCS Books (2012).

[10] Vos J Craig M Cooper M Existential therapies: a meta-analysis of their effects on psychological outcomes. J Consult Clin Psychol (2015) 83:115–28. 10.1037/a0037167 25045907

[11] Heidegger M SEIN UND ZEIT Elfte, unveränderte Auflage (1967), MAX NIEMEYER VERLAG TÜBINGEN.

[12] Heidegger M Being and Time. Translated by Joan Stambaugh (2010), State University of New York Press.

[13] Jones A Some reflections on clinical supervision: an existential-phenomenological paradigm. Eur J Cancer Care (Engl) (1998) 7:56–62. 10.1046/j.1365-2354.1998.00069.x 9582752

[14] Orbanic SD The Heideggerian view of person: a perspective conducive to the therapeutic encounter. Arch Psychiatr Nurs (1999) 13:137–44. 10.1016/S0883-9417(99)80044-2 10389342

[15] Moore LJ Goldner-Vukov M The existential way to recovery. Psychiatr Danub (2009) 21:453–62. 19935478

[16] Arimitsu K Development and validation of the Japanese version of the self-compassion scale. Jap J Psychol (2014) 85:50–9. 10.4992/jjpsy.85.50 24804430

[17] Neff KD Whittaker TA Karl A Examining the factor structure of the self-compassion scale in four distinct populations: is the use of a total scale score justified? J Pers Assess (2017) 99:596–607. 10.1080/00223891.2016.1269334 28140679

[18] Neff KD Tóth-Király I Yarnell LM Arimitsu K Castilho P Ghorbani N Examining the factor structure of the self-compassion scale in 20 diverse samples: support for use of a total score and six subscale scores. Psychol Assess (2019) 31:27–45. 10.1037/pas0000629 30124303

[19] Takahashi T Sugiyama F Kikai T Kawashima I Guan S Oguchi M Changes in depression and anxiety through mindfulness group therapy in Japan: the role of mindfulness and self-compassion as possible mediators. Biopsychosoc Med (2019) 13:4. 10.1186/s13030-019-0145-4 30820241 PMC6378713

